# Coordinating care for older adults in primary care settings: understanding the current context

**DOI:** 10.1186/s12875-018-0821-7

**Published:** 2018-08-07

**Authors:** Jacobi Elliott, Paul Stolee, Veronique Boscart, Lora Giangregorio, George Heckman

**Affiliations:** 1 University of Waterloo, 200 University Avenue West, Waterloo, ON N2L 3G1 Canada; 2 Conestoga College, 299 Doon Valley Dr, Kitchener, ON N2G 4M4 Canada

**Keywords:** Older adults, Primary care, Qualitative methods, Health care providers, Care coordination

## Abstract

**Background:**

It is well known that older adults are high users of the health care system. Older adults with chronic conditions receive care from multiple providers, across multiple settings, and this care is often unorganized and confusing. In 2005, Ontario established a model of inter-professional primary care (family health teams) with the aim of providing enhanced interdisciplinary primary care to patients. Primary care requires an in-depth understanding of the operations of primary care teams and their relationships with other community services. The aim of this study was to develop a deeper understanding of the current operations of two family health teams in Ontario, including their current processes for referrals, information sharing, and engagement of patients in decision-making.

**Methods:**

Focus group and individual semi-structured interviews with health care providers were conducted. Purposeful sampling was used to ensure information was obtained from different professional perspectives. Interviews were audio-recorded and transcribed verbatim. Using NVivo 10, data were analyzed using line by line thematic analysis techniques. A cluster technique was then applied to group similar codes into themes.

**Results:**

Three focus group interviews (involving 4–6 health care providers/focus group) and six individual interviews were conducted with health care providers from two primary care teams and surrounding community care organizations. Six key themes were identified: 1) challenges engaging older adults in decisions about their care; 2) who is responsible for coordinating the care? 3) fragmented information sharing between health care providers; 4) lack of standardized referral processes and follow-up; 5) identifying services in the community for older adults; and 6) caring for older adults in rural communities.

**Conclusions:**

The results of this study provide an in-depth understanding of the current context in which the primary care teams are currently operating. Improved primary care will require stronger processes of coordination, greater knowledge of and connections with other community services, and enhanced patient engagement processes. This information provides a helpful basis for implementing interventions in primary care.

**Electronic supplementary material:**

The online version of this article (10.1186/s12875-018-0821-7) contains supplementary material, which is available to authorized users.

## Background

Older adults use a large amount of health care services, however the current health care system is not well designed to meet their needs. Primary health care is seen as being the health care system “first point of contact” and as the patient’s medical “home”, helping patients navigate and coordinate their care journey [1, 2]. Effective primary health care also provides continuing care for chronic conditions and involves a wide range of health care providers in the care provided to patients [3].

Although primary health care would seem to be the best place within the system to provide and coordinate care for older adults, it is poorly positioned to do so. Through the development of both structural and funding-based barriers between primary and community care, the delivery of healthcare has become fragmented in many countries [4, 5]. Many patients, particularly those individuals who are older and who experience one or more chronic conditions, may require long-term, often complex care from multiple providers working in a variety of settings [6, 7]. There is a recognition that primary care needs to move away from a focus on episodic care in which the majority of patients seek attention for specific, acute complaints and leave care when treatment has been received [8]. Increasingly, health care providers are acknowledging the need to work together with patients, their families, and informal caregivers, and to collaborate with other health care providers to tailor healthcare to better fit the individual patient context [8].

Furthermore, family physicians are often considered to be the structural link for coordination between primary, community and hospital care for the patient [6]. Family physicians are viewed as the central medical professional in the care and management of chronic disease, in particular; in part, this is due to their longstanding relationship with the patient which allows them to take into account a longer term medical history and greater knowledge of the individual patient context [9]. However, most family physicians are not in a position to take on the duties of a full-time care coordinator. The demands of caring for patients with chronic conditions represents a substantial increase to physician workload [8]. Some primary health care practices have added or reorganized staff and delegated the work of coordination, creating a “care team”, who all participate actively in meeting the needs of an individual patient [8]. The idea of primary care “teams” was established in the Ontario, Canada health care context through the launch of Family Health Teams (FHTs) in 2005. FHTs are comprised of physicians, nurses and interdisciplinary care providers such as; social workers, dieticians, or occupational therapists, who provide services such as chronic disease management, counseling, education, and palliative care [10]. The use of care teams is said to improve efficiency, staff satisfaction, and the patient experience of care [8]. Although FHTs were established almost a decade ago, care is still disjointed between primary and community care in Ontario [10].

This study aimed to understand the current context and operation of primary health care teams, focusing on the current process for referring older adults to community care services from primary care; the current state of information sharing; and identification of services available to older adults in the community. This study is the first step of a larger research project where findings from this work will be used to guide implementation and evaluation of a model of care coordination for older adults in primary care. Specifically, this study aimed to answer the following question, what is the local context in which primary care teams were operating, including the available health and support services?

## Methods

Through a social constructivist perspective, this study used qualitative methods (focus group and individual interviews) and thematic analysis of the data to understand the current referral and care coordination process between primary care and community care in two primary care locations, a rural and an urban site.

Ethics clearance for this study was obtained from the University of Waterloo’s Office of Research Ethics (ORE #20452).

### Sampling and recruitment

Purposeful sampling was used to gather the perspectives of persons who might play a role in coordinating care for older persons (e.g. nurse, social worker, care coordinator). Two primary care sites were chosen and the community care organization that served seniors, working around that site were noted. It was also important to involve community care providers - those individuals who work outside of the primary care centre but receive referrals from the primary care centre. These could include community care coordinators, Alzheimer Society program directors, and care providers for community services such as nutrition services or transportation. Individuals were recruited through standardized email communication and participants were asked if they felt anyone else should be involved and contacted.

The target sample size for this phase of the study was approximately 6–8 individuals for each focus group, following common qualitative procedures [11]. Primary care provider and community care provider focus groups were kept homogenous as per common focus group approaches [11].

### Data collection

Focus group and individual interviews were conducted with primary care and community care providers. Interviews were conducted at two study sites, one representing a rural community and one an urban community. Focus group interviews were conducted with three groups: urban primary care team, urban community care representatives, rural community care representatives. Individual interviews were conducted with participants who could not attend focus group interviews, including rural primary care team providers. Focus group and individual interviews were conducted face-to-face or by telephone and lasted 60–90 min in length. These were audio-recorded and transcribed verbatim. In order to guide and assist the interviewer, an interview guide (Additional File 1) was created with questions that would enable the researcher to gain a better understanding of the current referral process, communication mechanisms, and information on how providers currently engage patients in care planning.

### Data Analysis

Data collected during the interviews consisted of verbatim transcripts for each focus group and individual interview. Using NVivo 10 software [12], all interview data were analyzed using line by line emergent coding as outlined by Lofland and colleagues [13]. Following a process outlined by Lofland and colleagues [13], initial coding was completed followed by focused coding where patterns, themes, and interesting concepts were identified. A clustering technique was completed, where similar codes were grouped into themes [13]. Each cluster was given a name and brief description, with quotations from the data to support the theme.

#### Ensuring Methodological Rigour

The use of member checking (i.e. sharing categories and interpretations with participants to determine if their realities are adequately represented) and development of an audit trail was used to promote trustworthiness of findings [14]. A process of reflexivity was also completed which allowed the researcher to be aware of their role throughout the project. Specifically, in this project, the researcher collected information from study participants, removing their own biases and allowed the experiences of the participants to be reflected in the results.

Results are presented in detail below, however some findings from this work are also presented in a local public document [15].

## Results

Three focus group interviews were completed with 4–8 participants in each group and six participants were interviewed individually (*n* = 30 participants in total). In total, representation from the rural community included six participants from primary care, six from community care, and two hospital representatives. Representation from the urban area included nine participants from primary care, six from community care, and one hospital representative. Community care providers represented organizations providing care including both community support services and home care services.

The findings revealed a number of themes related to the current primary and community care contexts. After reviewing the data and performing appropriate thematic analysis, six key themes emerged. Table 1 outlines the themes and subthemes.

**Table 1:** Themes and Subthemes

THEMES	Subthemes
1) *Challenges in Engaging Older adults in Decisions about their Care*	• *Older adults should be engaged more in decision-making than they are currently* • *Understanding why older adults decline services* • *“Time” is needed for meaningful conversations* • *Caregivers are an important part of the circle of care*
2) *Who is Responsible for Coordinating the care?*	• *The role of a coordinator* • *Role clarity needed among patients and providers* • *Primary health care as a hub for coordinating care*
3) *Fragmented Information Sharing between Health Care Providers*	• *Communication between primary care and community care is fragmented* • *Providers going beyond what is expected of them to get information about a client* • *Multiple documentation systems make it hard to access patient information*
4) *Lack of Standardized Referral Processes and Follow Up*	• *Types of referrals to community services* • *Issues with referring patients to external services*
5) *Identifying Services in the Community for Older Adults is challenging*	• *Many organizations offer a variety of services for older complex patients*
6) *Caring for Older Adults in a Rural Communities*	• *Cultural boundaries* • *Coordinating care in large geographical location*

### Challenges in engaging older adults in decisions about their care

Evident throughout many of the discussions was the fact that although healthcare providers felt that engagement of patients was important in health care decision-making, however either it was not done as well, or as often, as it should be, or some providers didn’t know how to engage patients in a meaningful way. During the interview, health care providers discussed how they currently involve older adults and their family caregivers in healthcare planning; the quotes below illustrate the various responses: quotes.

*Yes, we use surveys if that’s what you’re getting at … there’s really no participatory involvement in the care pathway or planning –* Primary Care Provider Urban.

*Not as much as they could or should be. I think that many feel powerless – not knowing what is available to them or how to ‘work the system’. –* Urban Community Care Provider.

Community providers felt that they offered their patients’ options but it was up to the patient to decide whether to accept the service. Providers felt that they couldn’t do anything if the patients declined services.

*We may present all of the options and make our suggestion about what would be most helpful, but ultimately if our client does not want that service then that’s up to them.* – Community Care Representative (Urban).

Providers recognized that as they get to know their patients better and build a relationship, discussions are open and patients may opt to participate in services.

*…as you build trust in the relationship, you can get people engaged in other services –* Community Care Provider (Urban).

Community care providers also identified issues when goals and preferences are not discussed with the patient. In the case below, the physician made a referral, however when the community care provider offered the service, the patient was not willing to accept the service. This could also point to limited engagement and education for the patient.

*We also want to have a better idea of what their goals are, because what the goal is, let’s say, for example, the goal for the family physician might be a med review by psychiatry. When we get in there we might have to do some work arounds, massaging that…and they [patient] may not be ready to say yes... So that sometimes, I think is a challenge….* – Community Care Provider (Rural).

Many providers acknowledged the benefits of engaging patients and families in health care decision-making but identified the challenges that go along with those discussions. In order to engage patients in a meaningful way, there is a level of education that needs to occur for both the providers and the patients. If the patients are not aware of the services that are being offered to them, they may not see the value and therefore may decline the service.

*I think it’s just a hard thing to have a menu and say what would you like, when they don’t really understand maybe fully what each service might bring them, so that needs to be explained when you’re offering this service or getting consent for a service –* Community Care Provider Rural.

Above all, a strong and trusting relationship is central to involving older adults in healthcare decision-making. However, the current Canadian healthcare system is not designed to support the time it takes to build a trusting relationship. As illustrated in the quote below, the health system also does not support the community care provider being the one touch point for the patient.

*It becomes challenging … it’s not efficient for me to drive every day to buy a coffee to drive over to [Bob] to sit for an hour talking about his cat. It’s not efficient, it’s not productive* per se*. So for those 15 to 20 times I have to drive to sit and talk about [Bob’s] cat, nothing comes out of that. But on the 21st time, I get buy-in. And then I get support for him. The other kind of caveat to that is because I try so hard and I go out of my way and I kind of bend over backwards to really focus on providing the client with that compassion and empathy that they really not ever experienced in their life, I become the person that they call for everything, which is also not efficient.* – Community Care Provider (Rural).

Overall, patient engagement was recognized as important, however providers stated that a) the system does not support meaningful engagement (e.g. time with patients); b) both providers and patients need to have more time and education around the services available to older adults in the community; and c) providers need more support for relationship building. This will allow for patients to express their goals and preferences and ultimately create a care plan that suits their individualized needs.

### Who is responsible for coordinating care?

During the focus group interviews, care coordination was discussed, including who providers felt was most responsible for coordinating care for older adults. Many participants agreed that care coordination should occur in the primary care setting where patients may have longer standing relationships with physicians and nurses. The following excerpts illustrate these views:

*Primary care should be the hub of care – and this requires coordination. I don’t think this is a new role, however. We have too many ineffective care coordinators throughout the system that are ‘system-centred’ rather than ‘client-centred’ – by that I mean that they coordinate the services that they are responsible for and connected to but no more. –* Community Care Provider (Urban).

The second quote also demonstrates the feelings expressed by some providers that there are already care coordinators in the system however, they work for an organization rather than working across the system to ensure a patient is connected to the right services. Regardless of where the coordination happens, having one person to communicate with is important for the patient:

*I think having one point of contact that people feel comfortable calling in to is essential. Like, you’ve got all of these other organizations out there, but especially our community here, being such a rural older population… they don’t like having to answer to an answering machine, they like a real voice. So I think having that real voice available to them is important for that system navigation piece. You know, have someone who’s going to say “Okay, this is where we need to go, and let’s make it happen.” –* Primary Care Provider (Rural).

Participants in each of the focus group discussions mentioned the important role of a system navigator. Participants debated whether there was a need for a specific navigator role. Others optimistically commented that the system could work collaboratively to coordinate care for an individual without a designated role.

*I would expect to see is that system navigation is part of a process or function of primary care and the home team.* – Community Care Provider (Urban).

*I think that people are not knowledgeable about programs and services until they need them, and then they’re in a crisis situation, and then it’s not a good time to be searching for information. So if there was someone they could contact then they’re not taking up primary healthcare time with a physician or nurse practitioner, over something that could be dealt with by the most appropriate service provider… I think by having a system navigator, that person could be followed and the right services put into place to prevent a serious fall or some other situation that people are picking up on –* Primary Care Provider (Urban).

It is also important to recognize that those patients who are familiar with the system and feel empowered to be close involved in their care may desire to take on the role as their own navigator. Having patients engaged in discussions would allow for the best decisions to be made for individual situations, as indicated.

*Trying to identify who is the most responsible person for coordinating care and that role may end up in all different places, depending upon the individual. So in some cases it might be someone in the community who has a long standing relationship with that person, in other cases it might be the CCAC*^1^ *care coordinator or perhaps if neither of those, it could be someone in primary care, but instead of having multiple people taking on, like ultimately someone has to take accountability, and it may not always be the same person –* Community Care Provider (Rural).

Many providers in the community felt that they were already coordinating care for older adults. However, even these individuals saw the importance of engaging primary care in discussions around patients.

*Well, I guess that, see that depends on what you’re asking for. Because if they have a requirement for the services that CCAC does, then we’re doing that kind of coordination of care piece, but having the primary care physician more engaged in those care plans would be ideal, and I don’t know if that happens all the time.* – Community Care Provider (Rural).

Although many agree that regardless of where the coordination role occurs, a dedicated individual should care for the patient and take on the responsibility of linking the individual to appropriate services. This raises the question of how feasible it is to dedicate one person to take on responsibility or have a role across the entire system.

*We’ve thought about that… It’s just whether the ministry agrees with it, right? So that’s the biggest thing… It’s a very important role for system navigation. I think some of the things we’ve put in place here… even though we don’t have a dedicated person doing that, we have a few people who do that role.* – Primary Care Provider (Rural).

### Fragmented information sharing among health care providers

When the topic of *information sharing among health care providers* arose during the focus group and individual interviews, many providers had much to say. Information sharing continues to cause challenges for providers across the entire health care system due in part to the many different electronic medical records (EMRs) being used. In the local health region in which this study was conducted, there are 13 different EMR systems used across the health care system (primary care, community care, hospitals, etc.). Many providers acknowledged this challenge,

*At this point what I think our biggest challenge is the whole lack of a common documentation system. Because I don’t have access to the client’s EMR, which is what doctors rely on for communication between their allied staff. So it’s very difficult to have a true sense of good collaboration because…the doctors especially really just don’t have the time to step away from the client to come and find me. And I may or may not be in the building. –* Primary Care (Rural).

Further to that, there are no standardized forms in place for communication between primary care and community care, creating fragmented, or often non-existent, communication between the two health sectors. Some health care providers have taken it upon themselves to come up with solutions to ensure the information is easily accessible for the physicians who do not have a lot of time to read multiple pages of a report. However, if every community care provider had their own method of reporting, this could cause more complication for a physician who may be trying to quickly review the document, as indicated in the excerpt below from the community care provider who recognizes her method for documenting may be more “detrimental” than helpful.

*Three of those four pages are information about a client’s name, marital status, and disease diagnosis. So the demographic information that occupies page one is really of no use to the doctor and after looking at page one think this is bananas and skip it. You know. They don’t have time. So what I try to do is send my note, I’ll do my home visit with the client and do my assessment and be very thorough in putting as much detail as I can about what happened when I was in the home. I really want the doctor to be able to read the note and feel like they were there. Sometimes that’s detrimental because sometimes doctors want a one sentence snapshot: Client is good, services are started, check, carry on. And when I send them this mass note that’s quite thorough, I’m not sure how many like or dislike that. –* Community Care Provider (Urban).

As demonstrated in the following participant quotations, many health care providers identified the need to improve information sharing among providers across the health care system.

*We um don’t have a lot of conversation going back and forth between primary care. What does happen sometimes … we would check on Clinical Connect*^2^ *to try and get more information about it which isn’t always helpful…sometimes they don’t give you all the information. Again, that’s limited too because Clinical Connect – not everybody is connected* – Community Care Provider (Urban).

*I think that’s an area that there’s a lot of room for growth and improvement on. It is, for us, it’s been more individualized, so as an example, if we know, if we’ve had a referral come from primary care locally and we’re working very closely around the care for an individual, there’s some natural systems in place to share and to communicate that back, but it really, really depends, we don’t have a standard, formal process for that, we talk about how that might happen…but it’s a bigger system to try to figure out how we communicate back. –* Community Care Provider (Urban).

Ultimately, these issues affect the patient as they are required to repeat their stories multiple times to multiple health care providers. Patients may lose trust in the system because they have already answered questions and don’t understand why the new health care provider hasn’t received that information.

*I agree. I agree. Yeah, no. Because they don’t like having to tell their story over and over and over again. The nice thing about here, with me doing kind of that role, is that I know everybody. And I have access to the EMR. So if they’ve told the story once, I can read through it, and I don’t need to ask them again. Right? So just having that peace of being able to tell it that once and that’s it. –* Primary Care Provider (Rural).

For providers that work within the same organization (or in rural settings), it may be more feasible to have a “quick hallway conversation” to discuss a patient. However, participants acknowledged the privacy issues surrounding those conversations. Participants also suggested that phone conversations should occur more often when a provider needs to get information about a patient.

*Call anybody, it’s okay to call…I’ve got this client right, and basically – just like what happened today... our hallway conversation: had a nurse come and say ‘[Provider Name], come here, I need to bounce something!’ so just having that and being able to do it… I know privacy is out there and I know there’s a lot of issues, but...*

The interview results indicate that information sharing is an issue across the entire health care system.

### Lack of standardized referral processes and follow-up

During the focus group and individual interview process, another theme emerged around the current process of appropriately referring patients to services in the community. Interviews with care providers made clear that presently, there is no standardized process for identifying which primary care patients would benefit from additional services.

*Interviewer: Do you currently use any standardized assessments on your older adults?*

*Primary Care Provider (Urban): No, definitely not on every older adult. Sometimes the Montreal Cognitive Health Assessment (MoCA) or screening for diabetes and hypertension. We go for the most commonly used ones.*

*Interviewer: And what about referral pathways for those patients, would most of your assessments have a referral piece to them as well?*

*Primary Care Provider (Urban): Not always, no.*

Care providers did discuss however, that when a patient was referred to a service, multiple modes of communication are used to make the referral including phone calls, fax, e-referrals, self-referral or referrals from friends and family,

*We do get referrals over the phone on our secure voicemail and by fax from various providers, generally they’re physicians, who do it that way, we also get referrals through the e-referral aspect as well* – Community Care Provider.

Health care organizations on the receiving end of the referral then need to keep track of the different referrals coming in through the various modes of communication. Multiple modes of communication make it challenging to track referrals and ensure patients are receiving the services they need.

In this particular health region, there is a centralized intake process for referring to specialized geriatric services. This allows a nurse who works for centralized intake to review the referral, access multiple databases of patient health information and put together a package that is sent on to the specialist. This helps to prioritize patient urgency as well provide information to the next health care provider who may not have access to all of the different EMR systems.

For some programs, there are a number of referrals made by friends and family on behalf of the patients, “*Phone calls, a majority of them are clients or family members” (Community Care Provider).* Sometimes health care providers discuss a service with the patient and then leave it up to the patient to access the service. One Family Health Team in the rural community indicated that they generally ask patients *“how confident are you feeling that you can make that call?”* and depending on the answer, providers may help make connections for that patient. Participants noted that patients can be informed about a service, but if they do not have someone helping to make the connection or attend with them, the referral may not follow through.

Another major issue discussed by multiple health care providers (participants) was the concern around offering programs or services to patients who needed the service but did not want to accept it.

*We’ll go out and visit people and a referral has been made, but really they don’t want the service and they don’t want any involvement…. but I also think, I mean the other part that I just also want to raise, is there are some people where the referrals don’t get made, and that to me is also an equally important issue.* – Community Care Provider (Urban).

Participants acknowledged these issues of patients declining services or people not being referred in the first place. Community care providers spoke about how important it was to educate primary care providers on the services that are offered in the community. Primary care providers tended to know about the few services that they referred to often.

### Identifying services in the community for older adults is challenging

During the focus group discussions with primary care providers at both study sites, it was evident that more education was needed in terms of what services were available in the community for older adults. Providers seemed to be familiar with the common services such as those offered by the Alzheimer Society or the Community Care Access Centre (CCAC) but they were not familiar with programs or services that could help older adults who were not yet considered frail. One provider summarized the issue well in the following comment,

*I think whoever is doing the referral or suggesting the referral, needs to be educated on services and resources and understand the system enough to say “here’s some of the options available to you” and “where would you like to start?” Because I think, you know, a care coordinator, for example, in the community, might see that there is a dementia that is starting with a client and feels that going to memory clinic might be a good option for the client, but says “but you can also see a specialist, that might be a good option too” knowing that the client is not able to access community very well, or there’s lots of other issues with the client, they may say “let’s start with outreach team, it’s a team of a care coordinator and specialized nurses that will look at your situation, do an assessment, talk to your physician about that assessment”, then maybe determine next steps, whether it’s seeing a geriatrician, so there’s always next steps. And it’s very individualized based on the client situation, so I think the person who is doing that assessment, be it the nurse, care coordinator, or family physician, is individuals deciding what might be the next steps based on what they know about the client –* Community Care Provider (Rural).

In order to create care plans that are appropriate for the patient, it would be important to be familiar with a wide range of services in the community that could be of benefit to older adults. Participants were asked to list the services to which they commonly referred, these included internal programs within the Family Health Teams such as diabetes management or nutrition classes, or external programs such as memory clinics, specialists’ referrals, or the CCAC. Subsequently, during the focus group discussions with community care providers, participants were asked to discuss services that their organizations offered that would be appropriate for older adults. The list generated from these discussions include transportation services, in home and community exercise programs, hearing clinics, arthritis education, meals on wheels, cooking class, friendship programs, support groups, and many more. Community care providers also identified that many of these services would be beneficial for older adults who are looking to maintain their health and independence in the community.

### Caring for older adults in a rural community

The last theme that emerged through the interviews was one related to caring for older adults in rural communities. Community care and primary care providers in the rural community discussed facilitators and challenges that they encounter when working with older adults. Within a rural community, providers talked about how “everyone knows everyone” both in terms of the patient knowing the care provider and care providers knowing each other. Both of these examples are illustrated in the excerpts below:

*I’m from this community and have been here for a number of years. So it makes it easier when I’m talking to these people, because they usually know me or my family. So that’s a big plus…they feel comfortable that way*. – Rural Primary Care Provider.

One community provider also discussed the importance of building a trusting relationship with patients. It is difficult to have patient buy-in without a long-standing relationship. New providers (within the rural community or from an urban organization) may be able to develop relationships with patients if they are connected through a provider with whom the patient already has an established relationship. This idea is further described in the two quotes below:

*The only other thing I would like to add is I think rural is unique, and I think that you know that the organizations in this community work very well together, and have trusting relationships with seniors, so often it’s someone from one of the core organizations in the rural townships that will then make the introduction to someone else and then the service will be accepted.* – Community Care Provider (Rural).

As illustrated above, older adults are concerned with their privacy and developing trust between the patient and provider is important.

One challenge encountered by rural community care providers is the large geographical area that they have to cover to provide services to older adults. Within one day, the community care provider could be driving long distances across the rural region to see different patients.

*It has been a real challenge, especially when I am one person who is working out of such a massive geography. –* Community Care Provider (Rural).

There are also limited care providers working in the region, so it may be that there is only one provider for the specific organization across the entire rural area.

Providing care for older adults in the rural community presents some unique challenges or differences. As discussed by the participants, relationships are key to the success of providing care to older adults, especially in rural communities where trust and privacy are a concern. Relationships between the patient and provider allow for greater buy-in by the patient to accept services, and relationships between providers allow for knowing who to contact when looking for specific services or information. The strengths and challenges identified above were not present in the urban setting. Although care providers in an urban setting may build a long-standing relationship with a patient, this is not the same as “everyone knows everyone” and therefore building the relationship takes time. Furthermore, community care providers are not working across large geographical regions, rather, they are responsible for small sub-sections of the urban region.

## Discussion

The aim of this paper was to understand the current state of primary care and the environment in which it operates, in relation to engaging patients in decision-making, referral processes and information sharing. Data were gathered from in-depth focus group and individual interviews with 30 health care providers in rural and urban centres. Data analysis yielded six key themes that allow for better understanding of the current context of primary care teams. Although many of the findings identified through this project were not entirely unfamiliar in health care system research, these results provide greater awareness of the current issues faced by the primary health care sector [2, 16–19]. Furthermore, the issues were echoed by multiple participants across different professions (nurse, physician, manager, etc.) and across different health care sectors (primary care and community care).

The first theme, *challenges in engaging older adults in health care decision making*, highlighted the importance of engaging older adults in care planning, however it was strongly identified that this is not what currently happens in daily practice. Previous studies have identified that caring for older adults is most effective in improving outcomes when it includes active in-person contact with patients and families [20]. Specifically, older adults living with multiple chronic conditions emphasize the need for convenient and flexible access to their healthcare providers, clear communication of plans for their care that are specific to their individual circumstances, and support from a care coordinator who is able to help in prioritizing their needs and who can also promote continuity in their care relationships [9, 20]. This is an area for improvement in primary care where longstanding relationships are already developed between patients and health care providers.

The second theme, *who is responsible for coordinating care?* and the third theme, fragmented *information sharing between health care providers*, both highlighted the current challenges experienced by health care providers when coordinating care for older adults. Participants felt that a specific coordinator role could be valuable however this role would not be of benefit to patients and providers unless it was supported by better engagement of patients and families in decision-making, as well continuity of care across the health system. Literature suggests that caring for and supporting patients and families includes utilizing the right services, at the right time, determined by level of complexity [18].

The fourth and fifth themes, *lack of standardized referral processes and follow-up, and identifying services in the community for older adults*, touched on current processes that occur when trying to organize services for older adults. Data indicated that multiple communication methods are used to make referrals. Furthermore, providers acknowledged they are unfamiliar with all of the services available in the community, and sometimes referrals to services are not made on behalf of the patients when they should be. These are areas for significant improvement to primary care for older adults. Literature demonstrates the need for multidisciplinary care for older frail adults because it significantly reduces fall risk, hospital use and nursing home admissions [21].

The final theme, *caring for older adults in rural communities,* identified some of the unique challenges and facilitators when providing care for older adults in rural communities including, community care providers working across large geographical regions, “everybody knowing everyone”, and the importance of building trusting relationships. Although there were some strengths of rural communities identified, such as providers knowing who to contact for services, there are significant challenges. There are not enough health care providers to adequately serve the number of older adults in rural communities [22]. Sometimes rural communities are described as wealthy in terms of social networks that can support older adults, however there is a lack of available community resources to support older adults who wish to age in their own home [22, 23]. The lack of access to specialists also poses a significant challenge for individuals who need geriatric support for complex health conditions.

### Limitations

There are two limitations of the study. First is the study recruitment process. The researcher used networks in the community to make connections with community care providers who might be interested in participating, limiting access to the smaller organizations who may not be as well known in the community. Secondly, the study is limited to only two sites within one Canadian province and therefore the results may not be representative of other primary care teams across Ontario or Canada. However, a rural and urban location were selected to illustrate two different contexts.

## Conclusions

This study has provided information that will be useful for the next phases of this larger research focus. This research identified issues experienced by providers when providing care to older adults and areas that future work should focus on to improve care coordination for older adults. This study resulted in more detailed understanding of the primary care environment as it relates to providing care to older adults. Specific issues related to caring for older adults were identified including: lack of involvement of older adults in care planning; trouble coordinating care across the system, including challenges with sharing and receiving information; and limited knowledge by primary care providers of appropriate services for individuals in the community. Based on these findings, further education is necessary for both providers and patients in terms of service availability as well as the importance of engagement in decision-making and how this can be achieved.

## Additional files

Additional file 1 A1. Health Care Provider Interview Guide. Interview and focus group guide which was used to conduct individual and focus group interviews in-person and over the phone. (DOCX 17 kb)

## Abbreviations

CCAC: Community Care Access Centre
EMR: Electronic Medical Record
FHT: Family Health Team
MoCA: Montreal Cognitive Health Assessment

## Footnotes

1 Community Care Access Centre (CCAC): There are 14 CCACs across Ontario funded through the Ontario Ministry of Health and Long-Term Care, CCACs coordinate home care services and long-term care placements.

2 Clinical Connect is a secure, web-based portal that provides physicians and other health care providers with access to patients’ EMR from hospitals and CCACs.
