# Supplementary material for: Coordinating care for older adults in primary care settings: understanding the current context
Source: BMC Fam Pract. 2018 Aug 7;19:137. doi: 10.1186/s12875-018-0821-7 (PMC6081869; doi:10.1186/s12875-018-0821-7)
Supplement: Supplementary file 1 — A1. Health Care Provider Interview Guide. Interview and focus group guide which was used to conduct individual and focus group interviews in-person and over the phone. (DOCX 17 kb) [file 12875_2018_821_MOESM1_ESM.docx]

# **Focus Group Questions with Health Care Providers**

1. Could you please tell me about your role/organization?
   - How long have you been working in your role?
2. Do you currently use any standardized assessments on your older patients?
3. We would like to get your thoughts and suggestions about how we can best implement the Assessment Urgency Algorithm into your clinic.
   - Ask about each of the intervention components – You have just learned about the AUA… What do you think the pathway should look like for someone at :
     - Low Risk
     - Medium Risk
     - High Risk
4. What are the foreseeable barriers to implementing the intervention components? How can we overcome these? What resources or supports do you need?
5. Are you currently referring older patients to community services?
   - ***If not*- Are you aware of any community services that could benefit older adults at low/medium/high risk levels?
   - ***If yes*- Are you aware of the adherence to community services referrals?
6. How do you current refer older patients to community services?
   - *Do you currently collaborate/ communicate with community services/ agencies that provide services to your older patients?
7. Can you tell me about how older persons and their families are engaged in setting goals or making decisions about their care?
